# Alpelisib-Induced Diabetic Ketoacidosis in a Patient With Metastatic Breast Cancer

**DOI:** 10.7759/cureus.19441

**Published:** 2021-11-10

**Authors:** Mohamad Abufaied, Unwam Jumbo, Adala Alqalalwah, Mohammad Khair Hamad

**Affiliations:** 1 Endocrinology and Diabetes, Hamad Medical Corportation (HMC), Doha, QAT; 2 Internal Medicine, Hamad Medical Corportation (HMC), Doha, QAT

**Keywords:** uncontrolled hyperglycemia, pi3k ( phosphatidylinosiotol-3-kinase ), breast cancer, alpelisib, dka

## Abstract

Alpelisib, a phosphatidylinositol-3-kinase (PI3K) inhibitor, is a new drug approved for metastatic breast cancer. Hyperglycemia is a known side effect of this medication, however diabetic ketoacidosis is rarely described. We are presenting a 64-year-old female with a known case of Type 2 diabetes mellitus (hemoglobin A1c [HbA1c] 5.6% ) controlled by metformin alone. She was also diagnosed with metastatic breast cancer. She received radiotherapy, trastuzumab and letrozole. Then, she was started on alpelisib as she failed other previous modalities. She presented to the emergency department with a two-week history of polyuria and polydipsia, and laboratory investigation results showed high anion gap metabolic acidosis, ketonemia, and hyperglycemia. She was treated for diabetic ketoacidosis (DKA). After the resolution of DKA, she was kept on daily insulin subcutaneous injections. She was restarted on a reduced dose of alpelisib, but despite this, her blood sugar readings continued to rise, requiring discontinuation of the medication with a resolution of hyperglycemia.

The goal of our case report is to emphasize the importance of close glucose monitoring when starting alpelisib to avoid serious complications like DKA.

## Introduction

Diabetic ketoacidosis (DKA) is a medical emergency characterized by hyperglycemia, metabolic acidosis, and ketonemia. It mainly occurs in patients with type 1 diabetes. Rarely, DKA may occur in patients with type 2 diabetes [1]. Breast cancer is the most frequently diagnosed cancer and the leading cause of cancer death in women worldwide [2]. Alpelisib is a newly FDA-approved drug that works as an active inhibitor of phosphatidylinositol-3-kinase (PI3K) alpha. It has been used along with fulvestrant in hormone receptor-positive, HER2-negative, PIK3CA-mutated, advanced breast cancer. Unfortunately, despite its proven efficacy, alpelisib is associated with multiple side effects. Hyperglycemia is one of the most common and severe adverse reactions [3]. We report this case to shine a light on DKA as a rare but severe complication of this new promising drug for advanced breast cancer.

## Case presentation

We present a 64-year-old female with a history of type 2 diabetes mellitus for more than 15 years. She was on metformin 1000 mg twice daily. Her glucose was perfectly controlled with the latest hemoglobin A1c (HbA1c) 5.6% without any hypoglycemia. She also had breast cancer with metastases to the liver and peritoneum. The tumor was estrogen receptor (ER)-positive, human epidermal growth factor receptor 2 (HER2)-negative, and positive for PI3K mutation. Her malignancy was progressing despite using multiple lines of treatment, including hormonal therapy. Due to the presence of PI3K mutation, alpelisib 300 mg plus fulvestrant 500 mg was initiated. The patient was not checking her blood glucose during the period when she was at home. After two weeks, the patient presented to the emergency department with a history of polyurea, worsening fatigue, and decreased appetite. She also complained of nausea and vomiting associated with abdominal pain for a few days.

**Figure 1:**
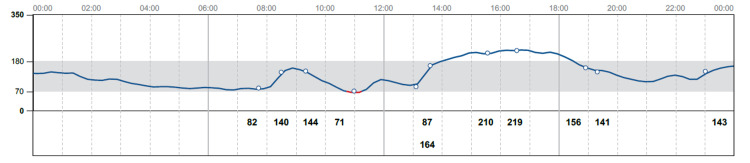
Blood glucose readings while patient was on insulin lispro and insulin glargine after diabetic ketoacidosis resolved

On admission, the patient was conscious and oriented. Her blood pressure was 143/76 mm Hg, her heart rate was 72 beats/minute, her temperature was 37 C, and her respiratory rate was 18 breaths/minute. Initial laboratory showed random blood glucose of 565 mg/dl, pH 7.191, anion gap 20 mmol/L, beta-hydroxybutyrate 4.05 mmol/bicarbonate 16 mmol/L, osmolality 316 mmol/Kg and lactate 1 mmol/L. The patient was admitted as a case of DKA, and she was started on insulin infusion and intravenous fluid for DKA management as protocol. She required a total of 144 units of intravenous insulin for 48 hours before being transitioned to a subcutaneous insulin regimen after resolution of DKA (Figure 1). Further laboratory tests showed C peptide 1.69 ng/ml and negative anti-glutamic acid antibodies. 

**Figure 2:**
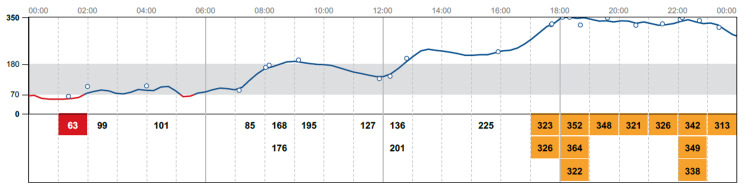
Rise in blood glucose readings when alpelisib was re-introduced at a lower dose (200 mg)

**Figure 3:**
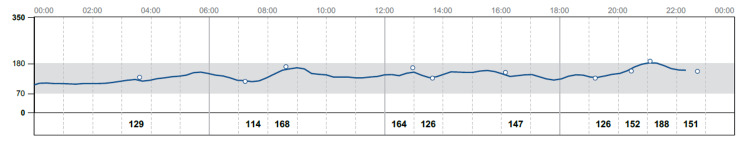
Controlled home blood glucose readings while on metformin

Her hospital blood glucose readings remained well controlled with multiple daily insulin injections. Alpelisib was restarted at a lower dose of 200 mg. Subsequently, blood sugar readings raised to a range between 280-320 mg/dl; however her metabolic panel was within normal range (Figure 2). Hereby, insulin doses increased to insulin lispro 12 units before each meal and 25 units insulin glargine, and alpelisib was discontinued. The patient was discharged home on insulin glargine at bedtime and insulin lispro before meals in addition to metformin. Continuous glucose monitoring tracing was done while she was at home. Blood glucose was within normal while insulin was gradually tapered down till stopped. She was kept on metformin, and her blood sugar readings remained within target (Figure 3).

## Discussion

Alpelisib works by inhibition of the PI3K pathway, which is important for regulation of cell processes and is the most frequently altered pathway promoting tumor growth, disease progression, and treatment resistance in HR+ advanced breast cancer [4,5]. The SOLAR-1 trial, a phase III randomized control trial, showed a more significant overall response in the cohort of PIK3CA-mutated cancer treated with alpelisib-fulvestrant as compared with placebo (26.6% vs. 12.8%) [3]. This is a profound breakthrough therapy but not without its delimiting adverse effects. The most recognized adverse effect in the phase III SOLAR-1 trial was hyperglycemia accounted for 63.7% of adverse effects reported, while only 0.7% of the total workgroup had DKA [3]. Alpelisib is said to interfere with the insulin signaling pathway leading to glycogen breakdown in the liver and preventing glucose uptake in skeletal muscle and adipose tissue. In patients with insulin resistance like in obese or type 2 diabetics, inhibition of PIK3CA using alpelisib may exacerbate hyperglycemia [6], and cause ketosis, as seen in our index patient who had well-controlled diabetes (HbA1c 5.6) on oral hyperglycemic agents (OHA). Furthermore, there have been five case reports published in the literature about alpelisib-induced DKA after the FDA approval [6-9].

Therefore, proper safety precautions need to be taken in initiating alpelisib, especially in diabetic patients due to the risk of DKA. The current manufacturer safety guidelines stipulate that before starting alpelisib, patients’ glucose should be optimized and fasting blood glucose monitored during therapy at least once a week for the first two weeks, then at least once every four weeks and as clinically indicated. Also required is HBa1c monitoring every three months and when indicated clinically. For those patients at risk of hyperglycemia, more frequent monitoring is required [10]. In patients with uncontrolled diabetes type 1 and 2, no safety data was reported as they were excluded due to the risk of increased morbidity [3]. The hyperglycemic effect of alpelisib is manageable usually by a dose modification (reduction, interruption, discontinuation) for grade 4 hyperglycemia (random blood sugar [RBS] >500 mg/dL or ≥27.8 mmol/L) and anti-hyperglycemic agent initiation or intensification depending on the grade of hyperglycemia that presents during therapy [10]. Most patients will usually reverse to normal glycemic control function after discontinuation of the medication. Standard OHA treatment and insulin are used for glycemic control as clinically indicated, with metformin having a specific dose incremental recommendation in the SOLAR-1 trial [10]. Our index patient, who initially had reasonable glycemic control on metformin, developed DKA and failed to control blood glucose despite being on incremental doses of insulin, ending with discontinuation of the medication.

## Conclusions

Although alpelisib has shown good outcome in patients with advanced breast cancer in terms of progression-free survival, hyperglycemia was a recognized reason for medication stoppage. We want to emphasize the importance of frequent blood glucose checking and optimization of diabetes medications to avoid hyperglycemia complications. We recommend closely monitoring patients with pre-existing diabetes who are at higher risk of hyperglycemia and DKA like in our index patient.
